# Motivation Profiles for Physical Activity Among Office Workers

**DOI:** 10.3389/fpsyg.2019.01577

**Published:** 2019-07-10

**Authors:** Tao Zhong, Hui Wang

**Affiliations:** ^1^ College of Sport and Health, Henan Normal University, Xinxiang, China; ^2^ College of Sport and Health, Zhejiang Sci-Tech University, Hangzhou, China

**Keywords:** physical activity, motivation, self-determination theory, latent profile analysis, person-centered approach

## Abstract

Physical activity is of importance for health enhancement. To promote physical activity involvement, motivation is considered to be a key factor. This study aimed to examine the motivation profiles for physical activity in a sample of Chinese office-based workers, grounded in a person-centered approach. Latent profile analysis was performed to generate motivation profiles for physical activity behavior. Successively, profile differences in relation to different motivations and physical activity were explored. Two motivation profiles emerged from the analysis. The two profiles differed significantly in various behavioral motivations and physical activity. The findings indicate that motivation profile characterized by autonomous motivation and introjected regulation is more favorable in physical activity participation, compared with a profile featuring external regulation and amotivation. The motivation profiles that naturally emerge are informative for future intervention design aiming to facilitate physical activity participation.

## Introduction

Health benefits received from participating in regular physical activity have been well documented (Rhodes et al., 2017). Despite the compelling evidence, a large number of adults fail to be physically active (Hallal et al., 2012). The condition is even worse for office-based workers, as they generally remain sedentary during working hours, which occupy a substantial amount of their waking time. Therefore, this sub-group of the adult population should be of particular concern in physical activity promotion campaigns. To date, motivation has been shown as an important psychosocial determinant for physical activity behavior (Quested et al., 2017). Self-determination theory (SDT), as a widely used motivational theoretical framework, may be useful to explain physical activity behavior. Unlike other motivation theories, SDT’s uniqueness lies in its emphasis on quality rather than quantity of motivation underlying behavior. It postulates that there are various types of motivations ranging along a continuum based on the degree of self-determination (Ryan and Deci, 2000). From the most to the least self-determined form, they are intrinsic motivation, integrated regulation, identified regulation, introjected regulation, external regulation, and amotivation (Deci and Ryan, 2008).

Intrinsic motivation indicates people who partake in an activity because of innate interest, or enjoyment of the activity *per se* (Ryan and Deci, 2000). Integrated regulation refers to engaging in a given behavior when it has been fully integrated within individuals, and the behavior is conceived congruent with individuals’ personal goals, values, or beliefs (Wilson et al., 2006). Identified regulation occurs when people are driven by personally valued outcomes accompanied with engaging in a certain behavior (Markland and Tobin, 2004). Introjected regulation, as another form of motivation, takes place when a behavior is regulated out of internal pressures, such as lessening guilt and shame, and facilitating self-esteem (Mullan et al., 1997; Gillison et al., 2009). With respect to external regulation, when directed by it, individuals engage in a certain behavior because of external demands imposed (Ryan and Deci, 2006). Finally, amotivation is a state of lacking any motivation to participate in a certain activity (Markland and Tobin, 2004).

Previous research has largely investigated the effect of a single behavioral motivation on physical activity, by adopting a variable-centered approach (Teixeira et al., 2012). Based on findings concerning the relation between various types of behavioral motivations and physical activity behavior, generally the positive effect of more autonomous forms of behavioral motivations including intrinsic motivation, integrated regulation, and identified regulation for physical activity behavior involvement has been revealed (Wininger, 2007; Silva et al., 2010), indicating the importance of fostering these types of behavioral motivations. As to introjected regulation and its link with physical activity behavior, generally findings are split into two categories, namely, a positive (Wininger, 2007) and a null association (Craike, 2008). While for external regulation, its negative relation (Ingledew and Markland, 2008) or null relation (Peddle et al., 2008) with physical activity behavior has been displayed. Finally, when it comes to the association between amotivation and physical activity behavior, generally a negative association is observed (Teixeira et al., 2012).

Though the above variable-centered approach can provide valuable information on the contribution of each single behavioral motivation to physical activity behavior, such an approach inevitably incurs the loss of information on how different behavioral motivations can be configured within an individual (Castonguay and Miquelon, 2017; Miquelon et al., 2017). Given that motivation should be regarded as a dynamic concept and people may hold multiple types of motivations simultaneously (Cox et al., 2013), the fact that previous studies heavily rely on the variable-centered approach will undermine our understanding on how different types of motivations can function together within an individual to drive people for physical activity (Friederichs et al., 2015). For instance, an individual may be motivated by both intrinsic motivation and identified regulation for physical activity participation, as the individual can engage in physical activity because of its innate pleasure, and personally endorsed accompanying outcomes (e.g., health improvement) in the meantime. To address the limitation, a person-centered approach is promising, because it has the theoretical advantage in helping us understand how various types of behavioral motivations may co-exist in different people (Moran et al., 2012). People with similar motivation configuration can be grouped together, forming a unique motivation profile. By locating potential motivation profiles in a population, a deeper understanding can be achieved on how people are motivated toward an activity (e.g., physical activity). Furthermore, from an application perspective, based on knowledge on motivation profiles, intervention can be more effective by tailoring to the need of a particular group of people who share a similar pattern of motivation profile (Friederichs et al., 2015).

To date, some attempts have been made to investigate motivation profiles for physical activity (Stephan et al., 2010; Guerin and Fortier, 2012; Ferrand et al., 2014; Friederichs et al., 2015; Castonguay and Miquelon, 2017; Miquelon et al., 2017). However, there are several limitations that can be noted from previous research, which provides opportunity for future research advancement. First, due to the motivation instrument employed, numerous studies do not involve the whole spectrum of various motivations in profiles construction. For instance, some studies use the Behavioral Regulation for Exercise Scale (BREQ), entailing intrinsic motivation, identified regulation, introjected regulation and external regulation, while omitting integrated regulation and amotivation, for constructing motivation profiles (Friederichs et al., 2015). Some other studies involve the use of the BREQ-2, which adds one additional motivation (i.e., amotivation) into the BREQ, in motivation profiles construction (Matsumoto and Takenaka, 2004; Guerin and Fortier, 2012; Ferrand et al., 2014; Castonguay and Miquelon, 2017). However, the instrument still lacks integrated regulation, as a type of behavioral motivation, in motivation profile construction. Very few studies have employed the BREQ-3, encompassing all behavioral motivations, particularly the integrated regulation, to inform motivation profiles construction (Miquelon et al., 2017). In fact, integrated regulation, as a highly internalized form of motivation, is suggested to be important for adults’ physical activity behavior (Wilson et al., 2006; McLachlan et al., 2011). Therefore, it will be meaningful to involve all the motivations, including the overlooked amotivation, and integrated regulation in particular, for determining people’s motivation profiles, which may provide a complete picture of people’s motivation profiles for physical activity.

Second, research grounded in a person-centered approach to examining physical activity motivation profiles is predominantly undertaken among individuals in western countries, while very few studies are conducted on individuals from non-Western countries (e.g., China). Therefore, findings on motivation profiles for physical activity in non-Western populations, such as in the Chinese adult population, are obscure. Research on this topic among the specific population is necessary, as past findings on motivational profiles for physical activity among Western samples cannot be simply assumed to be applicable and generalizable to other populations. The present research has the potential to bridge the knowledge gap by examining what motivational profiles for physical activity may emerge, and whether the motivational profiles among the target Chinese adult population may have their own features and differ from what has been found among people from Western countries. What is more, from a practical perspective, findings on physical activity motivational profiles among the target population can contribute to the knowledge base for future intervention design aiming to enhance their physical activity behavior. Third, from a methodological perspective, the traditional cluster analysis is the main statistical technique employed for research using the person-centered approach for motivation profile examination (Matsumoto and Takenaka, 2004; Boiché et al., 2008; Guerin and Fortier, 2012; Gillet et al., 2013; Ferrand et al., 2014). Cluster analysis is a statistical technique to group cases based on similarity and dissimilarity (Gore, 2000; Hair and Black, 2000). Despite its widely used position in prior research, this traditional strategy has received criticism because of its shortcomings such as the subjective nature associated with it (Pastor et al., 2007). To overcome its limitations, latent profile analysis, as a mixture model-based cluster analytic technique, has gained popularity in recent years in research using a person-centered approach (Gerber et al., 2014). Instead of clustering individuals using some arbitrarily chosen distance measures from a bottom-up approach (i.e., finding similarities among cases) as with the cluster analysis, latent profile analysis adopts a top-down approach (i.e., modeling the distribution of data) to deriving clusters using a probabilistic model (Pastor et al., 2007). It can provide statistics such as model fit indices to assess model fit and choose the optimal solution, which can better capture uncertainty in the classification process (Pastor et al., 2007). Thus, it may be advantageous to employ the latent profile analysis to assess potential physical activity motivation profiles for individuals.

In sum, the purpose of the present study was to examine the motivation profiles for physical activity in Chinese office-based workers *via* latent profile analysis and to investigate the potential differences across generated motivation profiles in relations to various behavioral motivations and physical activity behavior. Information on physical activity motivation profiles derived from the study can inform future intervention studies to optimize efficiency, by catering to the specific needs of people with different motivation profiles.

## Materials and Methods

### Participants and Procedure

The sample of the study involved 636 participants. They were office-based workers in China. The participants ages = 30.50 ± 5.13, ranged 20–60, with 249 males and 386 females (one participant did not specify gender). Of the total sample, a subsample (*N* = 504) completed a physical activity behavior measurement.

The study was carried out in accordance with the recommendations of Research Ethics Committee of College of Sport and Health, Henan Normal University. A research electronic survey, covered with an introduction, was created in a Chinese online survey platform^1^. In the introduction of the online survey, participants were informed the research purpose. Also, the anonymity, confidentiality, and voluntary basis of the research were underscored. Interested participants could access and complete the survey. Informed consent was obtained from the participants. The study protocol was approved by Research Ethics Committee of College of Sport and Health, Henan Normal University.

### Measures

#### Motivation Toward Physical Activity

Chinese version of the BREQ-3 was adopted in the present research. It incorporates the validated integrated regulation subscale (McLachlan et al., 2011) into the Chinese version of the BREQ-2 (Chung and Liu, 2012), which was translated and validated from the original English version (Markland and Tobin, 2004). The Chinese version of the integrated regulation subscale was generated through a translation and back-translation technique (Sperber, 2004). The Chinese BREQ-3 consists of 22 items, with intrinsic motivation subscale (four items), integrated regulation subscale (four items), identified regulation subscale (three items), introjected regulation subscale (three items), external regulation subscale (four items), and amotivation subscale (four items). Five-point Likert scale (from 1 = “not true for me” to “5 = very true for me”) is used to determine to what extent a particular item suits a participant. In the present study, the internal consistency reliability of each sub-scale of the Chinese BREQ-3 was: *α* = 0.78 for intrinsic motivation, *α* = 0.72 for integrated regulation, *α* = 0.60 for identified regulation, *α* = 0.69 for introjected regulation, *α* = 0.77 for external regulation, and *α* = 0.78 for amotivation. All the values of Cronbach’s alpha were greater than or equal to 0.60, which was comparable with previous results (Verloigne et al., 2011; Segatto et al., 2013). As to the validity of the scale, factor analysis provided some support for the model goodness-of-fit, where *χ*^2^(194) = 565.46, *p* < 0.001, *χ*^2^/*df* = 2.91, CFI = 0.90, TLI = 0.88, RMSEA = 0.055 (90% CI = 0.050–0.060), and SRMR = 0.06. The standardized factor loadings of all the items were larger than 0.55, with *p* < 0.001. Mean values, standard deviations, standardized factor loadings, and standard errors of the scale’s items are displayed in Table 1. Additionally, correlations among subscales of the Chinese BREQ-3 are reported in Table 2.

**Table 1:** Descriptive statistics, standardized factor loading, and standard error of the Chinese BREQ-3.

Subscale/item	Mean	SD	FL	SE
**Intrinsic motivation**				
Item 1	3.63	0.97	0.58	0.04
Item 2	3.91	0.93	0.75	0.03
Item 3	4.05	0.88	0.71	0.03
Item 4	4.05	0.85	0.73	0.03
**Integrated regulation**				
Item 1	3.95	0.90	0.59	0.03
Item 2	3.87	1.02	0.71	0.03
Item 3	3.69	0.96	0.61	0.03
Item 4	3.62	1.09	0.60	0.04
**Identified regulation**				
Item 1	4.59	0.70	0.57	0.06
Item 2	4.14	0.88	0.60	0.05
Item 3	4.38	0.75	0.57	0.05
**Introjected regulation**				
Item 1	3.09	1.17	0.66	0.03
Item 2	2.74	1.16	0.74	0.03
Item 3	3.06	1.07	0.57	0.04
**External regulation**				
Item 1	2.74	1.22	0.69	0.04
Item 2	2.88	1.16	0.68	0.04
Item 3	1.88	1.02	0.60	0.04
Item 4	2.56	1.23	0.72	0.03
**Amotivation**				
Item 1	1.68	0.98	0.76	0.04
Item 2	1.64	0.88	0.73	0.04
Item 3	1.40	0.75	0.63	0.05
Item 4	1.44	0.80	0.63	0.04

*SD, standard deviation; FL, standardized factor loading; SE, standard error*.

**Table 2:** Correlations among subscales of the Chinese BREQ-3.

	Intrinsic motivation	Integrated regulation	Identified regulation	Introjected regulation	External regulation
Integrated regulation	0.801^***^	–	–	–	–
Identified regulation	0.707^***^	0.754^***^	–	–	–
Introjected regulation	0.405^***^	0.716^***^	0.317^***^	–	–
External regulation	−0.165^**^	−0.056	−0.290^***^	0.245^***^	–
Amotivation	−0.560^***^	−0.501^***^	−0.935^***^	−0.091	0.474^***^

** *indicates significance at 99%*.

*** *indicates significance at 99.9%*.

#### Physical Activity Behavior

Participants’ physical activity behavior was assessed with the Global Physical Activity Questionnaire (GPAQ), developed by the World Health Organization (WHO). It has been widely adopted and its reliability and validity have been well established (Bull et al., 2009; Cleland et al., 2014). The 19 items GPAQ measures moderate and vigorous physical activity (MVPA) taking place in sub-domains including work, travel and recreation, lasting at least 10 min per bout. A score of MVPA is calculated by summating weekly time spent in moderate physical activity (with a weight of 1) and vigorous physical activity (with a weight of 2) across sub-domains in unit of minute (Powell et al., 2011).

### Data Analysis

Latent profile analysis was executed to obtain the optimal number of motivation profiles for physical activity *via* Mplus 7. Robust maximum likelihood estimation was employed. The analysis can best describe different categories of participants based on response patterns related with continuously measured variables (Geiser et al., 2014). Based on previous studies on a number of profiles for physical activity extracted (Matsumoto and Takenaka, 2004; Stephan et al., 2010; Guerin and Fortier, 2012; Ferrand et al., 2014; Friederichs et al., 2015; Castonguay and Miquelon, 2017; Miquelon et al., 2017), tentative two-, three-, and four-profile solutions were estimated and compared to explore the optimal plausible latent profile solution. For mode fit evaluation, it was assessed based on multiple indices (Tein et al., 2013). First, the Lo-Mendell-Rubin adjusted likelihood ratio test (LMRT) was used as an indicator for the optimal number of profiles that provide the best fit to the data. Specifically, it compares a target model with k profiles (e.g., a three-profile model) to a model with k-1 profiles (i.e., a two-profile model). *p* of the LMRT less than 0.05 indicates that the model with higher profile number is better. Second, Akaike information criterion (AIC) and the sample-sized adjusted Bayesian information criterion (aBIC) were introduced to ensure the best profile solution. Better model fit is determined by lower AIC and aBIC values (i.e., close to 0). Finally, the entropy criterion was also used. The entropy is an indicator reflecting how a certain profile solution accurately classifies individuals into their respective profiles. A higher value of the entropy indicates a better profile solution (i.e., close to 1.0). The indices thereof, together with substantive interpretation of profiles, would be considered jointly to determine the best profile solution (Geiser et al., 2014).

Successively, extracted profiles were compared in scores of subscales of the BREQ-3, encompassing intrinsic motivation, integrated regulation, identified regulation, introjected regulation, external regulation, and amotivation. It was anticipated that all the motivation variables used for building profiles would demonstrate statistically significant contribution. Moreover, physical activity level was compared across profiles to examine possible difference. ANOVAs in SPSS 22.0 were used for the above comparison analyses. Statistically significant level was set at 0.05. Eta-squared (*η*^2^) was used to represent effect size with the application of the following Cohen’s rule (Miles and Shevlin, 2001): small (0.01 ≤ *η*^2^ ≤ 0.05), moderate (0.06 ≤ *η*^2^ ≤ 0.13), and large (*η*^2^ ≥ 0.14).

## Results

**Figure 1:**
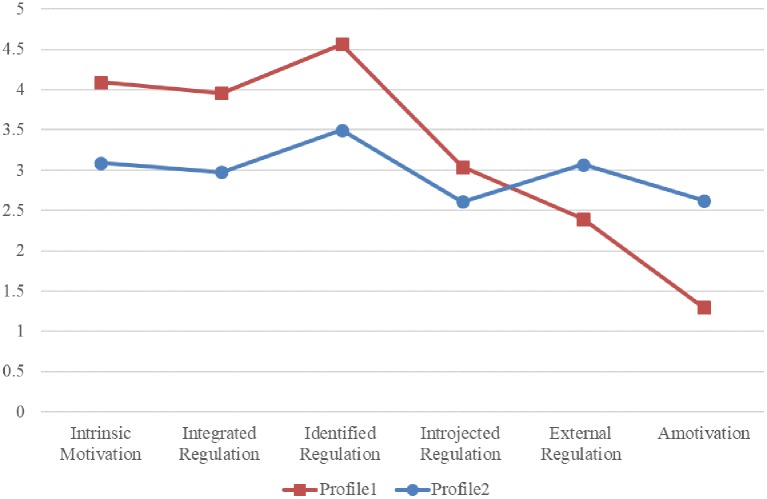
Scores of the two motivation profiles for physical activity.

The latent profile analysis revealed that a two-profile solution was superior to a one-profile solution, evidenced by a significant *p* of the LMRT. The three-profile solution, despite having slightly lower AIC and aBIC values, had the *p* of the LMRT insignificant, indicating it was not better than a two-profile solution. This result was further verified by the entropy value, as the three-profile solution had a lower value than did the two-profile solution, indicating inferior in classifying individuals. Given the statistical result and the readiness for a substantive interpretation of the solution, the two-profile solution was deemed the optimal model. The detailed statistical results are displayed in Table 3. The first profile consisted of 521 participants, while the second profile consisted of 115 participants. Based on the scores in individual behavioral motivation subscales of each profile, the first profile was named autonomous/introjected motivation profile, whereas the second profile was named external/amotivation profile. The motivation profiles for physical activity are illustrated in Figure 1.

**Table 3:** Model fit indices for the two-, three-, and four-profile solutions.

Fit indices	One-profile	Two-profile	Three-profile
LMRT	–	938.505, *p* < 0.001	247.422, *p* = 0.35
AIC	8506.068	7560.793	7321.895
aBIC	8521.431	7585.118	7355.182
Entropy	–	0.940	0.845

*AIC, Akaike information criterion; aBIC, sample size adjusted Bayesian information criterion; LMRT, Lo-Mendell-Rubin adjusted test*.

Successive ANOVAs showed that all the behavioral motivations contributed to latent profiles differentiation (*p* < 0.001), indicating the inclusion of the whole spectrum of behavioral motivations, including the somehow overlooked amotivation and especially the integrated regulation, for establishing physical activity motivation profiles, was meaningful. What is more, difference in physical activity behavior of the two motivation profiles was displayed. That is, the autonomous/introjected motivation profile was more favorable with respect to higher physical activity level, in comparison with the external/amotivation profile. The statistical results are shown in Table 4.

**Table 4:** Differences across motivation profiles.

	Profile 1 (*N* = 521)	Profile 2 (*N* = 115)	*p*	Eta-squared (*η*^2^)
Intrinsic motivation	4.09	3.08	<0.001	0.348
Integrated regulation	3.96	2.98	<0.001	0.296
Identified regulation	4.56	3.49	<0.001	0.656
Introjected regulation	3.04	2.60	<0.001	0.058
External regulation	2.40	3.06	<0.001	0.096
Amotivation	1.30	2.63	<0.001	0.690
Physical activity	177.57 ± 142.47	109.94 ± 147.52	<0.001	0.029

## Discussion

The current research sought to uncover physical activity motivation profiles among the relatively sedentary, office-based workers in China; and examine differences in various behavioral motivations and physical activity behavior across the established motivation profiles.

A person-centered approach was employed to investigate motivation profiles for physical activity in the present study. This approach is rooted in a holistic-interactionistic framework (Bergman and Andersson, 2010). Under this framework, an individual is considered as a coherent whole, with interactive elements working together to function as patterns of operating factors in a dynamic system (Bergman and Wångby, 2014). In a person-centered approach, “patterns of operating factors” is operationalized as how a set of variables can configure together to form specific patterns. The patterns are mutually exclusive and reflect characteristics of subgroups of individuals (Wångby-Lundh et al., 2018).

Previously, a different approach, the variable-centered approach has prevailed and been largely used in examining the association between behavioral motivations and physical activity behavior (Teixeira et al., 2012). From a methodological point of view, such an approach relies on linear statistical models in a correlation analysis (Bergman and Trost, 2006; Bergman and Andersson, 2010). While this approach can provide information concerning how different variables (behavioral motivations) can contribute to physical activity behavior, it views individuals as the summation of variables, which overlooks the possibility that an individual can be seen as an integrated totality (Bergman and Trost, 2006; Bergman and Andersson, 2010). In our study, through the construction of physical activity motivation profiles at the individual level by adopting a person-centered approach, a better understanding can be obtained regarding how different behavioral motivations can configure together at the individual level under a holistic-interactionistic framework. Furthermore, useful information can be derived in terms of how to design and perform physical activity interventions more efficiently by targeting people with different characteristics.

Based on the latent profile analysis, two distinct motivation profiles for physical activity emerged, namely the autonomous/introjected motivation profile and the external/amotivation profile. In addition to the advancement in analytical strategy employed (latent profile analysis rather than conventional cluster analysis), the current study extends previous research by taking all the behavioral motivations, including the relatively less used amotivation and especially the integrated regulation into account, to inform the construction of motivation profiles. Findings indicate that all the behavioral motivations contributed to produce a more complete picture of motivation profiles in the physical activity context.

The emergence of the specific pattern of two physical activity motivation profiles, from the office-based workers sample in China, differs from previous studies among western adult samples (Stephan et al., 2010; Guerin and Fortier, 2012; Friederichs et al., 2015; Castonguay and Miquelon, 2017; Miquelon et al., 2017). With regard to the autonomous/introjected motivation profile, it was relatively high in more autonomous motivation including intrinsic motivation, integrated regulation, identified regulation, and introject regulation, while relatively low in external regulation and amotivation. The result indicates that people with this motivation profile may hold intrinsic motivation, integrated, identified, and introjected regulations for physical activity simultaneously. It makes sense that intrinsic motivation, integrated and identified regulation configured together in the sub-group of people, as SDT posits that the three behavioral motivations are broadly considered as autonomous motivation. Therefore, the three behavioral motivations possess a relatively high level of self-determination, regardless of their specific reasons (e.g., enjoyment or valued outcome) for an activity engagement. It is interesting to note that the somewhat less self-determined behavioral motivation, namely, the introject regulation, also contributed to forming a distinct motivation profile, along with autonomous motivation. Introjected regulated behavior is initiated by guilt and shame avoidance, or by a feeling of self-worth (Gillison et al., 2009). Its combination with autonomous motivation to form a motivation profile might be under the influence of culture. In the Chinese culture, “face” is highly valued, and Chinese people are more inclined to avoid the risk of “losing face” and acknowledge the importance of “saving face” (Ting-Toomey et al., 1991; Li and Su, 2007). Thus, in the physical activity context, a Chinese individual may engage in physical activity because he or she fears a potential negative result such as a negative reaction from peers, which may lead to perceived manifestation of his or her failure and “losing face.” Such cultural values may significantly influence Chinese people’s emphasis on introjected regulation as a type of motivation for physical activity behavior. Thus, aside from autonomous motivation, such behavioral motivation contributed significantly to form a distinct motivation profile for physical activity, which is not observed in previous studies in western samples.

For the combination of external regulation and amotivation in the external/amotivation profile, it appears that people who are externally motivated for physical activity participation are also likely to be amotivated, which is characterized by lacking competence and failing to recognize the value of an activity (Markland and Tobin, 2004). Since externally regulated behavior is out of external pressures and demands (Markland and Tobin, 2004), and people with such a behavioral motivation may be unlikely to feel competent and value a behavior, it is not surprising to observe the two more controlled forms of motivations bind together to form a distinct motivation profile.

When it comes to the relation between motivation profiles and physical activity, results exhibited that people with the autonomous/introjected motivation profile were more physically active, which confirms the positive effect of autonomous motivation and introjected regulation on physical activity involvement (Stephan et al., 2010; Friederichs et al., 2015). Findings from the research highlight the positive effect of promoting autonomous motivation and introjected regulation in enhancing physical activity behavior among Chinese office-based workers.

Limitations and future directions should be addressed. First, a cross-sectional research design was employed, which limits the possibility to explore the longitudinal interplay between motivation profiles and physical activity behavior. Therefore, a longitudinal design in future research is advocated. Second, only volunteers participated in the present study, which may create a self-selection effect (Brown et al., 2017). Therefore, the generalization of the conclusion should be made with due caution. It is recommended that future research should aim to identify motivation profiles for physical activity among an enlarged representative sample. By doing so, some potentially unidentified sub-group of people may be better represented. Moreover, such research would offer an opportunity to inspect whether the two profiles located in the present study are replicable (Tanaka and Nolan, 2018). Third, self-reported physical activity measurement was adopted in the present study, namely the WHO’s GPAQ. Despite that the GPAQ has been proved to be a valid and reliable tool widely (Cleland et al., 2014), it still suffers from some biases such as recall bias (Baranowski, 1988; Sallis and Saelens, 2000). Therefore, objective measurements (e.g., accelerometer) should be considered to use. Finally, while the factorial validity and internal consistency reliability of the BREQ-3 in the Chinese office-based workers sample was overall established, it should be noted that the Cronbach’s alpha coefficient for the identified regulation subscale was relatively low (0.60). In a previous research involving Chinese adults, the value of the internal consistency reliability for the identified regulation subscale was 0.72 (Liu et al., 2015). However, lower values of the internal consistency reliability for the subscale were also reported in some previous research (Segatto et al., 2013). For instance, a study reported a value of the Cronbach’s alpha of 0.61 for the subscale (Verloigne et al., 2011). Therefore, there is some inconsistency in this aspect and the internal consistency reliability of the subscale seems less optimal. One possible reason might be to do with the limited number of items in the subscale (three items altogether). As stated in prior literature, Cronbach’s alpha, indicative of internal consistency reliability, is to some extent influenced by the number of items. A limited number of items in a scale (e.g., three) may increase the risk of compromising the internal consistency reliability of the scale (Cortina, 1993). Thus, the relatively low value of Cronbach’s alpha of the identified regulation subscale, indicative of its less optimal internal consistency reliability, may be in part ascribed to the subscale’s limited number of items. Future research may consider revising the subscale, such as by incorporating newly designed and validated items into the subscale and testing whether the internal consistency reliability of the subscale can be improved.

To summarize, the present research reveals there are two motivation profiles for physical activity among Chinese office-based workers. The profiles characterized by intrinsic motivation, integrated, identified, and introjected regulations, are more favorable than the profiles characterized by external regulation and amotivation, in relation to physical activity participation. In light of these findings, physical activity practitioners should not emphasize the use of external contingences (e.g., punishment or reward) to promote physical activity. Even though SDT research has acknowledged that external regulation can be useful in initial behavior uptake, it may jeopardize long-term physical activity adherence. Instead, it is advocative of fostering an autonomy supportive environment for physical activity behavior, as it is conceived to be beneficial for internalization of behavioral motivation and can facilitate more self-determined behavioral motivation (Teixeira et al., 2012). What is more, different intervention strategies can be employed to tailor to people with different types of motivation profiles, which in turn can boost intervention efficiency. For example, for those who have been moderately autonomously motivated, they can benefit from reinforcing their autonomous motivation. As for individuals with the external/amotivation profile, they can gain from exploring goals that are related to personal core values. All in all, useful information can be provided for physical activity intervention design based on information of motivation profiles.

## Ethics Statement

The study was carried out in accordance with the recommendations of Research Ethics Committee of College of Sport and Health, Henan Normal University. In the introduction of an online survey used, participants were informed the research purpose. Also, the anonymity, confidentiality, and voluntary basis of the research were underscored. Interested participants could access and complete the survey. Informed consent was obtained from the participants. The study protocol was approved by Research Ethics Committee of College of Sport and Health, Henan Normal University.

## Author Contributions

TZ conducted the study and drafted the manuscript. HW participated in the design of the study and helped revise the manuscript. The authors read and approved the final manuscript.

### Conflict of Interest Statement

The authors declare that the research was conducted in the absence of any commercial or financial relationships that could be construed as a potential conflict of interest.

## Footnotes

^1^https://www.wjx.cn/

## References

[ref1] Baranowski T. (1988). Validity and reliability of self report measures of physical activity: an information-processing perspective. Res. Q. Exerc. Sport 59, 314–327. 10.1080/02701367.1988.10609379

[ref2] Bergman L. R. Andersson H. (2010). The person and the variable in developmental psychology. J. Psychol. 218, 155–165. 10.1027/0044-3409/a000025

[ref3] Bergman L. R. Trost K. (2006). The person-oriented versus the variable-oriented approach: are they complementary, opposites, or exploring different worlds? Merrill-Palmer Q. 52, 601–632. 10.1353/mpq.2006.0023

[ref4] Bergman L. R. Wångby M. (2014). The person-oriented approach: a short theoretical and practical guide. Est. J. Edu. 2, 29–49. 10.12697/eha.2014.2.1.02b

[ref5] Boiché J. Sarrazin P. G. Grouzet F. M. E. Pelletier L. G. Chanal J. P. (2008). Students’ motivational profiles and achievement outcomes in physical education: a self-determination perspective. J. Educ. Psychol. 100, 688–701. 10.1037/0022-0663.100.3.688

[ref6] Brown J. L. C. Ong J. Mathers J. M. Decker J. T. (2017). Compassion fatigue and mindfulness: comparing mental health professionals and MSW student interns. J. Evid. Inf. Soc. Work 14, 119–130. 10.1080/23761407.2017.1302859, PMID: 28388339

[ref7] Bull F. C. Maslin T. S. Armstrong T. (2009). Global physical activity questionnaire (GPAQ): nine country reliability and validity study. J. Phys. Act. Health 6, 790–804. 10.1123/jpah.6.6.790 20101923

[ref8] Castonguay A. Miquelon P. (2017). Motivational profiles for physical activity among adults with type 2 diabetes and their relationships with physical activity behavior. Health Psychol. Behav. Med. 5, 110–128. 10.1080/21642850.2016.1272416

[ref9] Chung P. K. Liu J. D. (2012). Examination of the psychometric properties of the Chinese translated behavioral regulation in exercise questionnaire-2. Meas. Phys. Educ. Exerc. Sci. 16, 300–315. 10.1080/1091367X.2012.693364

[ref10] Cleland C. L. Hunter R. F. Kee F. Cupples M. E. Sallis J. F. Tully M. A. (2014). Validity of the global physical activity questionnaire (GPAQ) in assessing levels and change in moderate-vigorous physical activity and sedentary behaviour. BMC Public Health 14:1255. 10.1186/1471-2458-14-1255, PMID: 25492375 PMC4295403

[ref11] Cortina J. M. (1993). What is coefficient alpha? An examination of theory and applications. J. Appl. Psychol. 78, 98–104. 10.1037/0021-9010.78.1.98

[ref12] Cox A. E. Ullrich-French S. Sabiston C. M. (2013). Using motivation regulations in a person-centered approach to examine the link between social physique anxiety in physical education and physical activity-related outcomes in adolescents. Psychol. Sport Exerc. 14, 461–467. 10.1016/j.psychsport.2013.01.005

[ref13] Craike M. (2008). Application of self-determination theory to a study of the determinants of regular participation in leisure-time physical activity. World Leis. J. 50, 58–69. 10.1080/04419057.2008.9674527

[ref14] Deci E. L. Ryan R. M. (2008). Self-determination theory: a macrotheory of human motivation, development, and health. Can. Psychol. 49, 182–185. 10.1037/a0012801

[ref15] Ferrand C. Martinent G. Bonnefoy M. (2014). Exploring motivation for exercise and its relationship with health-related quality of life in adults aged 70 years and older. Ageing Soc. 34, 411–427. 10.1017/S0144686X12001092

[ref16] Friederichs S. A. H. Bolman C. Oenema A. Lechner L. (2015). Profiling physical activity motivation based on self-determination theory: a cluster analysis approach. BMC Psychol. 3:1. 10.1186/s40359-015-0059-2, PMID: 25678981 PMC4310201

[ref17] Geiser C. Okun M. A. Grano C. (2014). Who is motivated to volunteer? A latent profile analysis linking volunteer motivation to frequency of volunteering. Psychol. Test Assess. Model. 56, 3–24. 10.1037/t00898-000

[ref18] Gerber M. Jonsdottir I. H. Lindwall M. Ahlborg G. Jr. (2014). Physical activity in employees with differing occupational stress and mental health profiles: a latent profile analysis. Psychol. Sport Exerc. 15, 649–658. 10.1016/j.psychsport.2014.07.012

[ref19] Gillet N. Vallerand R. J. Paty B. (2013). Situational motivational profiles and performance with elite performers. J. Appl. Soc. Psychol. 43, 1200–1210. 10.1111/jasp.12083

[ref20] Gillison F. Osborn M. Standage M. Skevington S. (2009). Exploring the experience of introjected regulation for exercise across gender in adolescence. Psychol. Sport Exerc. 10, 309–319. 10.1016/j.psychsport.2008.10.004

[ref21] Gore P. A. (2000). “Cluster analysis” in Handbook of applied multivariate statistics and mathematical modeling. eds. Tinsley H. E. A. Brown S. D. (San Diego: Academic Press), 297–321.

[ref22] Guerin E. Fortier M. (2012). Motivational profiles for physical activity: cluster analysis and links with enjoyment. Rev. phénEPS/PHEnex J. 4, 1–21. 10.1176/appi.ajp.162.6.1237

[ref23] Hair J. Black W. (2000). “Cluster analysis” in Reading and understanding MORE multivariate statistics. eds. Grimm L. G. Yarnold P. R. (Washington, DC: American Psychological Association), 147–205.

[ref24] Hallal P. C. Andersen L. B. Bull F. C. Guthold R. Haskell W. Ekelund U.. (2012). Global physical activity levels: surveillance progress, pitfalls, and prospects. Lancet 380, 247–257. 10.1016/S0140-6736(12)60646-1, PMID: 22818937

[ref25] Ingledew D. K. Markland D. (2008). The role of motives in exercise participation. Psychol. Health 23, 807–828. 10.1080/08870440701405704, PMID: 25160882

[ref26] Li J. J. Su C. (2007). How face influences consumption – a comparative study of American and Chinese consumers. Int. J. Mark. Res. 49, 237–256. 10.1177/147078530704900207

[ref27] Liu J. D. Chung P. K. Zhang C. Q. Si G. (2015). Chinese-translated behavioral regulation in exercise questionnaire-2: evidence from university students in the Mainland and Hong Kong of China. J. Sport Health Sci. 4, 228–234. 10.1016/j.jshs.2014.03.017

[ref28] Markland D. Tobin V. (2004). A modification to the behavioural regulation in exercise questionnaire to include an assessment of amotivation. J. Sport Exerc. Psychol. 26, 191–196. 10.1123/jsep.26.2.191

[ref29] Matsumoto H. Takenaka K. (2004). Motivational profiles and stages of exercise behavior change. Int. J. Sport Health Sci. 2, 89–96. 10.5432/ijshs.2.89

[ref30] McLachlan S. Spray C. Hagger M. S. (2011). The development of a scale measuring integrated regulation in exercise. Br. J. Health Psychol. 16, 722–743. 10.1348/2044-8287.002009, PMID: 21199546

[ref31] Miles J. Shevlin M. (2001). Applying regression and correlation. A guide for students and researchers. London: Sage.

[ref32] Miquelon P. Chamberland P.-É. Castonguay A. (2017). The contribution of integrated regulation to adults’ motivational profiles for physical activity: a self-determination theory perspective. Int. J. Sport Exerc. Psychol. 15, 488–507. 10.1080/1612197X.2016.1155637

[ref33] Moran C. M. Diefendorff J. M. Kim T.-Y. Liu Z.-Q. (2012). A profile approach to self-determination theory motivations at work. J. Vocat. Behav. 81, 354–363. 10.1016/j.jvb.2012.09.002

[ref34] Mullan E. Markland D. Ingledew D. K. (1997). A graded conceptualisation of self-determination in the regulation of exercise behaviour: development of a measure using confirmatory factor analytic procedures. Personal. Individ. Differ. 23, 745–752. 10.1016/S0191-8869(97)00107-4

[ref35] Pastor D. A. Barron K. E. Miller B. J. Davis S. L. (2007). A latent profile analysis of college students’ achievement goal orientation. Contemp. Educ. Psychol. 32, 8–47. 10.1016/j.cedpsych.2006.10.003

[ref36] Peddle C. J. Plotnikoff R. C. Wild T. C. Au H.-J. Courneya K. S. (2008). Medical, demographic, and psychosocial correlates of exercise in colorectal cancer survivors: an application of self-determination theory. Support Care Cancer 16, 9–17. 10.1007/s00520-007-0272-5, PMID: 17569994

[ref37] Powell K. E. Paluch A. E. Blair S. N. (2011). Physical activity for health: what kind? How much? How intense? On top of what? Public Health 32, 349–365. 10.1146/annurev-publhealth-031210-101151, PMID: 21128761

[ref38] Quested E. Ntoumanis N. Thøgersen-Ntoumani C. Hagger M. S. Hancox J. E. (2017). Evaluating quality of implementation in physical activity interventions based on theories of motivation: current challenges and future directions. Int. Rev. Sport Exerc. Psychol. 10, 252–269. 10.1080/1750984X.2016.1217342

[ref39] Rhodes R. E. Janssen I. Bredin S. S. D. Warburton D. E. R. Bauman A. (2017). Physical activity: health impact, prevalence, correlates and interventions. Psychol. Health 32, 942–975. 10.1080/08870446.2017.1325486 28554222

[ref40] Ryan R. M. Deci E. L. (2000). Intrinsic and extrinsic motivations: classic definitions and new directions. Contemp. Educ. Psychol. 25, 54–67. 10.1006/ceps.1999.1020 10620381

[ref41] Ryan R. M. Deci E. L. (2006). Self-regulation and the problem of human autonomy: does psychology need choice, self-determination, and will? J. Pers. 74, 1557–1586. 10.1111/j.1467-6494.2006.00420.x, PMID: 17083658

[ref42] Sallis J. F. Saelens B. E. (2000). Assessment of physical activity by self-report: status, limitations, and future directions. Res. Q. Exerc. Sport 71, 1–14. 10.1080/02701367.2000.11082780 25680007

[ref43] Segatto B. L. Sabiston C. M. Harvey W. J. Bloom G. A. (2013). Exploring relationships among distress, psychological growth, motivation, and physical activity among transplant recipients. Disabil. Rehabil. 35, 2097–2103. 10.3109/09638288.2013.807882 23829354

[ref44] Silva M. N. Markland D. Vieira P. N. Coutinho S. R. Carraça E. V. Palmeira A. L. (2010). Helping overweight women become more active: need support and motivational regulations for different forms of physical activity. Psychol. Sport Exerc. 11, 591–601. 10.1016/j.psychsport.2010.06.011

[ref45] Sperber A. D. (2004). Translation and validation of study instruments for cross-cultural research. Gastroenterology 126, S124–S128. 10.1053/j.gastro.2003.10.016 14978648

[ref46] Stephan Y. Boiché J. Le Scanff C. (2010). Motivation and physical activity behaviors among older women: a self-determination perspective. Psychol. Women Q. 34, 339–348. 10.1111/j.1471-6402.2010.01579.x

[ref47] Tanaka R. Nolan R. P. (2018). Psychobehavioral profiles to assist tailoring of interventions for patients with hypertension: latent profile analysis. J. Med. Internet Res. 20:e149. 10.2196/jmir.8757 29752248 PMC5970280

[ref48] Tein J. Y. Coxe S. Cham H. (2013). Statistical power to detect the correct number of classes in latent profile analysis. Struct. Equ. Model. 20, 640–657. 10.1080/10705511.2013.824781, PMID: 24489457 PMC3904803

[ref49] Teixeira P. J. Carraça E. V. Markland D. Silva M. N. Ryan R. M. (2012). Exercise, physical activity, and self-determination theory: a systematic review. Int. J. Behav. Nutr. Phys. Act. 9:78. 10.1186/1479-5868-9-78 22726453 PMC3441783

[ref50] Ting-Toomey S. Gao G. Trubisky P. Yang Z. Soo Kim H. Lin S.-L. (1991). Culture, face maintenance, and styles of handling interpersonal conflict: a study in five cultures. Int. J. Confl. Manag. 2, 275–296. 10.1108/eb022702

[ref51] Verloigne M. De Bourdeaudhuij I. Tanghe A. D’Hondt E. Theuwis L. Vansteenkiste M. (2011). Self-determined motivation towards physical activity in adolescents treated for obesity: an observational study. Int. J. Behav. Nutr. Phys. Act. 8, 1–11. 10.1186/1479-5868-8-97 21923955 PMC3189862

[ref52] Wångby-Lundh M. Klingstedt M. L. Bergman L. R. Ferrer-Wreder L. (2018). Swedish adolescent girls in special residential treatment: a person-oriented approach to the identification of problem syndromes. Nord. Psychol. 70, 17–46. 10.1080/19012276.2017.1323663

[ref53] Wilson P. M. Rodgers W. M. Loitz C. C. Scime G. (2006). “It’s who I am… really!” The importance of integrated regulation in exercise contexts. J. Appl. Biobehav. Res. 11, 79–104. 10.1111/j.1751-9861.2006.tb00021.x

[ref54] Wininger S. R. (2007). Self-determination theory and exercise behavior: an examination of the psychometric properties of the exercise motivation scale. J. Appl. Sport Psychol. 19, 471–486. 10.1080/10413200701601466

